# Use of the checkerboard DNA-DNA hybridization technique for bacteria detection in *Aedes aegypti *(Diptera:Culicidae) (L.)

**DOI:** 10.1186/1756-3305-4-237

**Published:** 2011-12-20

**Authors:** Analiz de Oliveira Gaio, Rivea CC Rodrigues, Cássio do Nascimento, Nagila FC Secundino, Francisco JA Lemos, Paulo FP Pimenta, Nadia Monesi

**Affiliations:** 1 Laboratório de Biotecnologia, Universidade Estadual do Norte Fluminense-UENF, Av. Alberto Lamego 2000, Horto, 28013-602, Campos dos Goytacazes, RJ, Brazil; 2 Departamento de Materiais Dentários e Prótese, Faculdade de Odontologia de Ribeirão Preto - USP, Av. do Café, s/n, Monte Alegre, 14040-904, Ribeirão Preto, SP, Brazil; 3 Laboratório de Entomologia Médica, Centro de Pesquisas René Rachou, Fundação Oswaldo Cruz, Av. Augusto de Lima 1715, 30190-002, Barro Preto, Belo Horizonte, MG, Brazil; 4 Departamento de Análises Clínicas, Toxicológicas e Bromatológicas, Faculdade de Ciências Farmacêuticas de Ribeirão Preto - USP, Av. do Café s/n, Monte Alegre, 14040-903, Ribeirão Preto, SP, Brazil

**Keywords:** Checkerboard DNA-DNA hybridization, Aedes aegypti, bacteria

## Abstract

**Background:**

Bacteria associated with insects can have a substantial impact on the biology and life cycle of their host. The checkerboard DNA-DNA hybridization technique is a semi-quantitative technique that has been previously employed in odontology to detect and quantify a variety of bacterial species in dental samples. Here we tested the applicability of the checkerboard DNA-DNA hybridization technique to detect the presence of *Aedes aegypti*-associated bacterial species in larvae, pupae and adults of *A. aegypti*.

**Findings:**

Using the checkerboard DNA-DNA hybridization technique we could detect and estimate the number of four bacterial species in total DNA samples extracted from *A. aegypti *single whole individuals and midguts. *A. aegypti *associated bacterial species were also detected in the midgut of four other insect species, *Lutzomyia longipalpis, Drosophila melanogaster*, *Bradysia hygida *and *Apis mellifera*.

**Conclusions:**

Our results demonstrate that the checkerboard DNA-DNA hybridization technique can be employed to study the microbiota composition of mosquitoes. The method has the sensitivity to detect bacteria in single individuals, as well as in a single organ, and therefore can be employed to evaluate the differences in bacterial counts amongst individuals in a given mosquito population. We suggest that the checkerboard DNA-DNA hybridization technique is a straightforward technique that can be widely used for the characterization of the microbiota in mosquito populations.

## Findings

The identification of bacteria in mosquito guts has relied on both culture-dependent and culture-independent techniques [1-3]. Molecular techniques for bacterial identification have received particular attention because they are more rapid than traditional culture methods and in addition can detect bacteria that cannot be cultured. Culture independent methods have mainly been based on the amplification of the 16S rRNA genes by PCR, followed by the identification of the amplified genes through nucleotide sequence comparisons [4].

The checkerboard DNA-DNA hybridization technique [5-8] is a semi-quantitative technique that has been extensively employed in odontology to detect and quantify a variety of bacterial species in dental samples and allows the simultaneous analysis of a large number of DNA samples against a range of DNA probes from different bacterial species on a single support membrane [8]. Here we have tested if this technique is suitable to detect and estimate the number of bacteria in total DNA samples extracted from both whole *Aedes aegypti *and from dissected *A. aegypti *midguts. In addition, we have also tested if we could detect and estimate the numbers of *A. aegypti *midgut-associated bacteria species in the midgut of other insect species.

In our experiments we employed a modified version [9] of the original DNA-DNA hybridization technique [8] (Additional file 1). As probes we used whole genomic DNA extracted from four bacterial species. *Serratia sp. *(FJ372764), *Asaia sp. *(FJ372770) and *Klebsiella sp. *(FJ372760) were isolated from laboratory-bred *A. aegypti *[1,2]. *Chryseobacterium sp. *(EU169680.1) was isolated from wild-caught *A. aegypti*.

**Figure 1:**
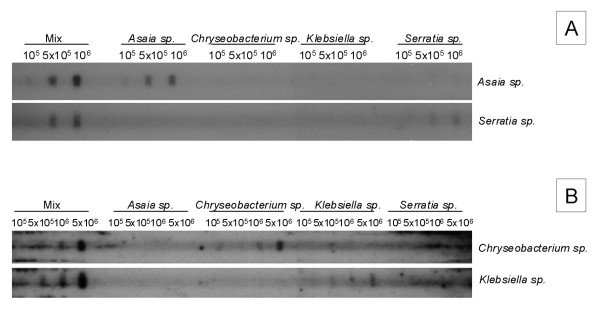
**Specificity of the genomic DNA probes**. Vertical lanes contained genomic DNA amounts equivalent to different numbers of cells of each tested species (10^5^, 5 × 10^5^, 10^6^, 5 × 10^6^, as indicated). The samples labeled "Mix" are standards that contain DNA amounts equivalent to 10^5^, 5 × 10^5^, 10^6^, 5 × 10^6^, cells of each tested species. (A) The horizontal rows contain either *Asaia sp. *or *Serratia sp. *genomic DNA probes previously diluted in hybridization buffer, as indicated, and (B) the horizontal rows contain either *Chryseobacterium sp. *or *Klebsiella sp. *genomic DNA probes previously diluted in hybridization buffer, as indicated. A signal at the intersection of the horizontal and vertical lanes indicates the presence of a species.

The results obtained after the hybridization of the phosphatase alkaline-labeled bacterial probes with defined amounts of total genomic DNA extracted from each bacterial species are shown in Figure 1. As can be observed, signals of increasing intensity are observed after the hybridization of defined amounts of the *Asaia sp. *and *Serratia sp. *probes with DNA amounts equivalent to 1 × 10^5^, 5 × 10^5 ^and 1 × 10^6 ^cells of these two species (Figure 1A). Similar results are observed after the hybridization of the *Klebsiella sp. *and *Chryseobacterium sp. *probes with DNA amounts equivalent to 1 × 10^5^, 5 × 10^5^, 1 × 10^6 ^and 5 × 10^6 ^cells of these two species (Figure 1B). Together, these results reveal that the intensity of the signals is proportional to the amounts of DNA immobilized on the membranes, and further show that the genomic probes are specific and only detect the corresponding genomic DNAs. The sensitivity of our protocol, which enabled the detection of DNA amounts ranging from 10^5 ^to 10^6 ^cells, is similar to that described both in the original checkerboard DNA-DNA hybridization protocol [8] and in the modified versions [5,9].

**Figure 2:**
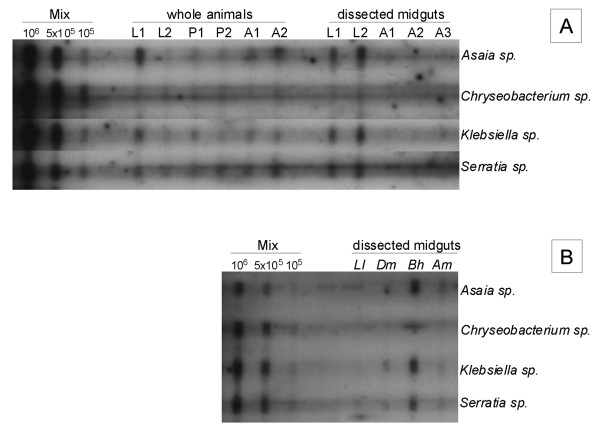
***A. aegypti *midgut associated bacteria are detected both in whole animals and in dissected midguts**. A. Analysis of DNA extracted from *A. aegypti*. Vertical lanes contain genomic DNA extracted from single whole fourth instar larvae (L1, L2), single whole old pupae (P1, P2), single whole sucrose-fed adult females (A1, A2), single midguts dissected from fourth instar larvae (L1, L2) and single midguts dissected from sucrose-fed adult females (A1, A2, A3). B. Analysis of DNA extracted from single midguts of *L. longipalpis *(*Ll*), *D. melanogaster *(*Dm*), *B. hygida *(*Bh*) and *A. mellifera *(*Am*). The horizontal rows contained the indicated DNA probes diluted in hybridization buffer, as indicated in the right hand side. The samples labeled "Mix" contain amounts of DNA equivalent to 10^5^, 5 × 10^5 ^and 10^6 ^cells of each tested species.

To test if this technique could detect the presence of bacterial species in *A. aegypti *samples, a membrane containing total genomic DNA extracted from single whole fourth instar larvae, old pupae, sucrose-fed adults and total genomic DNA extracted from dissected fourth instar larval and sucrose-fed adult midguts was hybridized to the four bacterial probes (Figure 2A). Different amounts of bacterial cells were present in the different *A. aegypti *samples (Table 1). *Asaia sp.*, *Klebsiella sp. *and *Serratia sp. *were present in amounts of > 10^5 ^and < 6 × 10^5 ^cells in whole larvae, old pupae and adults and in larval midguts, with the exception of one whole larva (L2) and one pupa (P2), in which amounts < 10^5 ^cells of both *Asaia sp. *and *Klebsiella sp. *cells were detected (Table 1). The detection of *Asaia sp. *and *Serratia sp. *in larvae, pupae and adults of *A. aegypti *is consistent with previous studies that showed strong interactions between these bacteria and mosquito species [1,10,11]. In addition, even though a statistical analysis could not be performed due to the small number of samples investigated, our experiments suggest the presence of generally higher amounts of cells in *A. aegypti *larvae (whole larva L1; midguts L1 and L2) as compared to whole pupae (Figure 2A and Table 1). These results corroborate with other studies showing a reduction in bacterial numbers after the transition from the last feeding larval stage to the pupal stage [12]. On the other hand, lower amounts (<10^5 ^cells) of *Chryseobacterium sp. *cells were detected in whole larvae, pupae, adults and in larval midguts. These results might explain why this bacterial genus has not been identified in *A. aegypti *[1,2], despite its high prevalence in *Anopheles gambiae *[13]. Finally, in adult midguts the investigated species were either not detected or detected at counts < 10^5 ^(Table 1). The higher number of bacterial cells detected in whole adults as compared to that observed in adult midguts could be attributed to bacterial colonization of other *A. aegypti *tissues as has been demonstrated for both *A. aegypti *and *Anopheles stephensi *[10,14].

**Table 1:** Estimated numbers of bacterial cells in whole animals and dissected midguts

Insect samples	*Asaia sp.*	*Chryseobacterium sp.*	*Klebsiella sp.*	*Serratia sp.*
*A. aegypti *L1 (w)	5.7 × 10^5^	<10^5^	3.2 × 10^5^	1.8 × 10^5^
*A. aegypti *L2 (w)	<10^5^	<10^5^	<10^5^	1.1 × 10^5^
*A. aegypti *P1 (w)	1.6 × 10^5^	<10^5^	1.4 × 10^5^	1.5 × 10^5^
*A. aegypti *P2 (w)	<10^5^	<10^5^	<10^5^	1.5 × 10^5^
*A. aegypti *A1 (w)	2.5 × 10^5^	<10^5^	1.2 × 10^5^	1.8 × 10^5^
*A. aegypti *A2 (w)	3.4 × 10^5^	<10^5^	1.4 × 10^5^	3.1 × 10^5^
*A. aegypti *L1 (mg)	3.0 × 10^5^	<10^5^	2.8 × 10^5^	2.0 × 10^5^
*A. aegypti *L2 (mg)	5.1 × 10^5^	<10^5^	4.4 × 10^5^	2.9 × 10^5^
*A. aegypti *A1 (mg)	< 10^5^	<10^5^	<10^5^	N.D.
*A. aegypti *A2 (mg)	N.D.	N.D.	<10^5^	<10^5^
*A. aegypti *A3 (mg)	N.D.	N.D.	<10^5^	N.D.
*L. longipalpis *(mg)	N.D.	N.D.	<10^5^	<10^5^
*D. melanogaster *(mg)	<10^5^	N.D.	2.1 × 10^5^	<10^5^
*B. hygida *(mg)	1.4 × 10^6^	N.D.	8.1 × 10^5^	7.2 × 10^5^
*A. mellifera *(mg)	3.1 × 10^5^	N.D.	1.6 × 10^5^	<10^5^

The images shown in Figure 2 were digitized and the numbers of bacterial cells in individual whole *A. aegypti *and dissected insect midguts of *A. aegypti*, *Lutzomyia longipalpis, Drosophila melanogaster*, *Bradysia hygida *and *Apis mellifera *were estimated using the Image-Quant TL software (GE Healthcare UK), as described in Additional file 1.

w, whole individual; mg, midgut; ND, not detected.

Our results show that the checkerboard DNA-DNA hybridization technique can be employed to detect the presence of bacterial species known to be associated with *A. aegypti *in *A. aegypti *samples. This technique reveals differences in the counts of bacteria present in distinct life stages and is sensitive enough to detect differences in the amount of bacterial cells amongst individual samples [for example, Figure 2A, whole larvae (L1 and L2) hybridized to the *Asaia sp. *probe]. Overall, our results demonstrate that the checkerboard DNA-DNA hybridization is a suitable technique for routine investigation of mosquito samples.

The presence of these four bacterial species was also investigated in midguts dissected from another insect vector, *Lutzomyia longipalpis*, and from three other insect species *Drosophila melanogaster*, *Bradysia hygida *and *Apis mellifera *(Figure 2B, Table 1). *Klebsiella sp. *and *Serratia sp. *were both detected in all four insect species tested. *Asaia sp. *cells were detected in *D. melanogaster*, *A. mellifera *and *B. hygida. Chryseobacterium sp. *was the only bacterial species not detected in this group of insects. *Klebsiella sp. *and *Serratia sp. *have been previously reported in *D. melanogaster, A. mellifera *and *L. longipalpis *[15-18]. In addition, our results revealed the presence of *A. aegypti *midgut-associated bacteria species in the midgut of *B. hygida*, an insect species in which the indigenous microbiota has not previously been characterized.

The use of the checkerboard DNA-DNA hybridization technique to detect and estimate bacteria from insects is appealing since it can contribute to the characterization of insect microbiota without the need of employing culture dependent methods that are both laborious and time consuming. Sample preparation is simple, which enables the rapid and simultaneous investigation of numerous samples collected from distinct populations. In addition, this method has the sensitivity to detect bacteria in single individuals at different developmental stages (larval, pupal), as well as in a single organ such as the midgut, and therefore, can be employed to determine if there are differences amongst individuals in a single population. Finally, the use of this technique can contribute to the characterization of the microbial ecology associated with mosquitoes, elucidate intrinsic and extrinsic factors that influence bacterial composition and identify the bacteria that are implicated in vectorial capacity differences between mosquito populations.

## Competing interests

The authors declare that they have no competing interests.

## Authors' contributions

AOG, RCCG, NS and NM performed the experiments. AOG and CN performed the quantification of the results. FJAL, PFPP and NM designed the study and drafted the manuscript. All authors read and approved the final manuscript.

## Supplementary Material

Additional file 1 **Experimental procedures**. The file provides a detailed description of the experimental procedures employed. Click here for file
