# Supplementary material for: Use of the checkerboard DNA-DNA hybridization technique for bacteria detection in Aedes aegypti (Diptera:Culicidae) (L.)
Source: Parasit Vectors. 2011 Dec 20;4:237. doi: 10.1186/1756-3305-4-237 (PMC3265427; doi:10.1186/1756-3305-4-237)
Supplement: Additional file 1 — Experimental procedures. The file provides a detailed description of the experimental procedures employed. [file 1756-3305-4-237-S1.PDF]

Additional file 1

Experimental procedures

In our experiments we employed a modified version [1] of the original DNA-DNA hybridization technique [2]. Briefly, the original protocol employed a labeling and detection system from Roche in which probes were labeled with digoxigenin. After hybridization and washes the blots were incubated with anti-digoxigenin antibody and the signal was detected by chemiluminescence using the Lumi-Phos™530 [2]. The modified version used in this work employed the AlkPhos Direct labeling and Detection System from GE Healthcare in which probes are directly labeled with a thermostable alkaline phosphatase enzyme. After hybridization and washes, hybrids are detected by chemiluminescence using the Gene Images CDP-*Star* reagent.

Bacterial DNA extraction

Genomic DNA was extracted using DNAzol® (Invitrogen, USA), following the manufacturer's instructions, from the following bacterial species: *Serratia sp.* (FJ372764), *Asaia sp.* (FJ372770), *Klebsiella sp.* (FJ372760) and *Chryseobacterium sp.* (EU260135.1). In order to confirm the identity of the isolates, initially each DNA was employed as a template for the amplification of the 16S rRNA intergenic region, followed by the sequencing of the PCR product. Nucleotide sequence comparisons were then performed in public databases (GenBank), which confirmed the identity of each genomic DNA.

Probe labeling

One hundred nanograms of genomic DNA from each species were directly labeled with a thermostable alkaline phosphatase enzyme using the AlkPhos Direct Labeling and Detection System (GE Healthcare UK). Probes were adjusted to a final

concentration of 1 ng/µL and stored at -20 ºC in 50 % glycerol. Calibration experiments were performed to determine the probe concentrations that allowed the quantification of DNA amounts corresponding to $1x10^5$, $5x10^5$ and $1x10^6$ cells of the tested species to optimize the signal to noise ratio.

Sample preparation immobilization onto nylon membranes

The external surface of the insects was washed and sterilized according to [3]. Individual whole *A. aegypti* samples and dissected insect midguts from *A. aegypti*, *Lutzomyia longipalpis, Drosophila melanogaster*, *Bradysia hygida* and *Apis mellifera* were transferred to a microtube and homogenized using 150 µl of TE buffer (10 mM Tri-HCl, 1 mM EDTA, pH 7.6), followed by the addition of 150 µl of 0.5 M NaOH, and boiling for 5 min. The samples were centrifuged at 15000 *g* for 10 min, and DNA was precipitated from the supernatants by adding 800 µl of 5 M ammonium acetate. The DNA samples were applied in parallel lanes onto nylon membranes using a Minislot device (Immunetics, Cambridge, Massachusetts), and fixed by baking for 2h, at 80 °C. As standard controls, defined amounts of genomic DNA corresponding to either $1x10^5$, $5x10^5$ and $1x10^6$ bacterial cells of the tested species were prepared, denatured, precipitated and applied to three control lanes.

Hybridization and signal detection

The membranes were prehybridized at 60 °C, for 16 hours, in hybridization buffer (GE Healthcare UK) containing 1 M NaCl and 4% blocking reagent (wt/vol). Defined amounts of DNA probes were placed on the membrane at right angles to the DNA sample lanes using of a Miniblotter apparatus (Immunetics, Cambridge, Massachusetts). Hybridizations were performed at 60 °C, for 16 hours, under gentle agitation. The membranes were subsequently washed twice at 65 °C, for 30 min in primary wash buffer [150 mM NaCl, 1 mM $MgCl_2$, 2 M urea, 0.1% sodum dodecyl sulfate, 0.2% blocking reagent (wt/vol), 50 mM NaH_2PO_4, pH 7.0] and twice in secondary wash buffer (100 mM

NaCl, 2 mM $MgCl_2$, 50 mM Tris-HCl, pH 10.0), at room temperature for 15 min. After washing, the hybrids were detected by chemiluminescence using the Gene Images CDP-*Star* Reagent (GE Healthcare UK). The signals were detected by exposing the membranes to ECL Hyperfilm- MP (GE Healthcare UK), and the images were digitized followed by analysis with the Image-Quant TL software (GE Healthcare UK). The pixel intensities in the control lanes, which contained DNA amounts corresponding to known cell numbers, were compared to the pixel intensities in the experimental lanes and employed as references to determine the number of cells of each bacterial species in the insect samples.

1. do Nascimento C, de Albuquerque RF, Jr., Monesi N, Candido-Silva JA: **Alternative method for direct DNA probe labeling and detection using the checkerboard hybridization format.** *Journal of clinical microbiology* 2010, **48:**3039-3040.
2. Socransky SS, Smith C, Martin L, Paster BJ, Dewhirst FE, Levin AE: **"Checkerboard" DNA-DNA hybridization.** *Biotechniques* 1994, **17:**788-792.
3. Gusmão DS, Santos AV, Marini DC, Russo ES, Peixoto AMD, Bacci MJ, Berbert-Molina MA, Lemos FJA: **First isolation of microorganisms from the gut diverticulum of *Aedes aegypti* (Diptera: Culicidae): new perspectives for an insect-bacteria association.** *Mem Inst Oswaldo Cruz* 2007, **102:**919-924.
